# ELK1 regulates BMPR1B transcriptional activity in ovine granulosa cells

**DOI:** 10.3389/fcell.2025.1623135

**Published:** 2025-07-01

**Authors:** Anwar Abdurahman, Yuling Ga, Xuehai Ma

**Affiliations:** ^1^ Xinjiang Key Laboratory of Mental Development and Learning Science, School of Psychology, Xinjiang Normal University, Urumqi, Xinjiang, China; ^2^ College of Animal Science and Technology, Nanjing Agricultural University, Nanjing, China

**Keywords:** Elk1, BMPR1B, transcription factor, granulosa cells, hu sheep

## Abstract

BMPR1B, a type I receptor in the BMP/Smad signaling pathway, was the first major gene identified in sheep (*Ovis aries*) to regulate key reproductive traits such as ovulation rate (OR) and litter size (LS). Despite its critical role in reproductive performance, the transcriptional regulatory mechanisms governing ovine BMPR1B expression remain poorly understood. This study identified the promoter region of the BMPR1B gene and revealed that transcription factor ELK1 regulates its transcriptional activity. Luciferase reporter assays identified the region from −438 bp to −208 bp relative to the essential promoter of the BMPR1B gene as a critical regulatory element. Notably, candidate ELK1-binding elements (EBS) were detected in this promoter region. Interestingly, ELK1 significantly enhances BMPR1B transcriptional activity by binding to specific sites in the promoter region. Further analysis in ovine granulosa cells showed that ELK1 modulates BMPR1B expression and influences granulosa cell apoptosis through the BMPR1B signaling pathway. These findings provide new key targets and mechanistic insights into the molecular regulatory network of ovarin granulosa cell apoptosis, advancing our understanding of reproductive biology in sheep.

## 1 Introduction

The BMP/Smad signaling pathway is crucial for reproduction in females and animals, as it governs essential functions like the development of ovarian follicles, the process of ovulation, and the implantation of embryos (Kumar et al., 2020). Within the ovary, oocytes release BMP ligands such as BMP15 and GDF9, which influence granulosa cells to enhance the processes of folliculogenesis and oocyte maturation by modulating cell growth and differentiation (Shimasaki et al., 2004). This route additionally affects the choice and supremacy of follicles by increasing sensitivity to FSH, a key factor in the growth of robust follicles (Knight and Glister, 2006). In the process of ovulation, the interplay of BMP/Smad signaling with the LH signaling route (Mukai et al., 2007) is crucial for the successful breaking of follicles and the liberation of oocytes. The signaling pathway involving BMP/Smad is critical within the uterus, as it supports endometrial receptivity and the process of decidualization, which are essential for the successful attachment of an embryo (Monsivais et al., 2021). Furthermore, this route is crucial for the development of the placenta, encompassing the differentiation of trophoblasts and the development of blood vessels, both of which are critical for sustaining a healthy pregnancy (Monsivais et al., 2021; Liao et al., 2024). In the realm of animals, the BMP/Smad signaling mechanism is essential, with mutations in BMP-related genes, including BMP15 and GDF9, correlating with modified ovulation rates and reproductive capabilities in agricultural species. Disruption of this biological pathway may result in reproductive issues like polycystic ovary syndrome and untimely ovarian dysfunction, underscoring its importance in female fertility and animal reproduction (Galloway et al., 2000; Persani et al., 2010). Members of the BMP/Smad signaling pathway are widely expressed in ovarian tissue (Yang et al., 2021; Wei et al., 2023). In sheep, the BMP/Smad signaling pathway is closely related to fertility, BMP15, BMPR1B, and GDF9 are the major genes for sheep reproductive traits, and there is a relationship between their expression levels in ovarian tissue and fertility. (Xu et al., 2010; Abdurahman et al., 2019). In sheep of the same breed, the gene expression levels associated with the BMP/Smad signaling pathway in the ovaries vary between those with high fertility and those with low fertility (Xu et al., 2010). The study found that in the ovarian tissue of Holstein sheep in the high fertility group, the mRNA expression levels of GDF9, BMP4, BMPR1B, BMPR2, and Smad4 genes in the antral follicles were significantly higher than those in the low fertility group (Abdurahman et al., 2022). In GMM sheep, the mRNA levels of BMP4, BMP7, GDF5, GDF9, BMP15, BMPR1A, and BMPR2 in the ovarian tissues of sheep (type BB) carrying the high fertility allele FecB of BMPR1B gene were significantly higher than those of non-carriers (type ++). The mRNA levels of Smad1, Smad4, Smad5, Smad8 and BMPR1B were significantly lower than those of non-carriers (++)Type) (Bahire et al., 2019). The protein Elk-1 is a member of the ETS family and features an ETS domain as a transcription factor, which is essential for modulating gene expression in reaction to different cellular signals. ELK1 is mainly recognized for its role in the MAPK/ERK signaling cascade, where it undergoes phosphorylation and activation through ERK (Sogut et al., 2021; Thiel et al., 2021). After being activated, ELK1 moves into the nucleus where it attaches to particular DNA sequences called ETS binding sites, influencing the transcription of genes that contribute to the process of cell differentiation, cell growth, and programmed cell death. Our previous report showed that in ovine granulosa cells, the transcriptional activity of BMPR1B is enhanced by the feedback mechanism of Smad4 (Abdurahman et al., 2019). However, there are few studies on the expression and regulation of the BMPR1B gene in sheep ovarian tissue. In this research, we found that ELK1 binds to the essential promoter of the BMPR1B gene 5′regulatory region, thereby increasing the transcription level of the gene.

## 2 Materials and methods

### 2.1 Sample collection

The ear tissue of Hu sheep was obtained from Xilaiyuan Breeding Farm (Taizhou, China) and was used for DNA extraction. Fresh sheep ovaries were collected from the Hualing slaughterhouse (Urumqi, Xinjiang) and used for the isolation and culture of ovarian granulosa cells. The Animal Care and Use Committee at Nanjing Agricultural University reviewed and authorized the animal research, which was carried out following the Experimental Animal Administration Regulations (China, Decree No. 2 from the State Science and Technology Commission, approved on 14 November 1988, approval code: SYXK 2017-0027).

### 2.2 Cell culture and transfection

The KGN and HEK293T cell lines were purchased from the Shanghai Cell Bank of the Chinese Academy of Sciences for Dual-Luciferases assay, the specific experimental steps were performed based on the method characterized in our previous study (Regan et al., 2016). Fresh ovaries were sourced from the Hualing Slaughterhouse in Urumqi, China, to culture ovine granulosa cells. They were maintained in sterilized saline at a temperature of 37°C and delivered to the laboratory within 2 h. Following the previously established protocol (Du et al., 2018), granulosa cells were isolated and maintained in culture.

### 2.3 Nucleic acid extraction and qRT-PCR

The traditional phenol-chloroform technique was employed to isolate DNA. The extraction of total RNA from the granulosa cells and ovarian follicles of sheep was performed using the TRIzol reagent provided by Invitrogen, located in Carlsbad, California, United States. Following that, cDNA was generated with the aid of a Prime Script™ RT Master Mix produced by TaKaRa, Dalian, China. Using a kit (SYBR Premix Ex Taq, Takara), the qRT-PCR was executed in triplicate, with GAPDH serving as the internal standard for comparison. The relative expression levels of target genes were assessed using the method. The 2^−ΔΔt^ method was utilized to measure the expression levels of the genes of interest.

### 2.4 Vector construction

The expression vector pcDNA3.1-ELK1, which encodes ELK1, was created in our laboratory. To determine the main promoter of the BMPRIB gene in Hu sheep, we amplified the 5′upstream sequence using a method based on deletion expression. The PCR products were cleaned up with the Axy prepTM PCR cleanup kit (Axygen, Union City, CA, United States) and subsequently inserted into the pGL3 luciferase reporter vector, which was then introduced into *E. coli* to create the luciferase reporter plasmids. Using an Endo-free plasmid mini kit II50 (Omega, Norcross, GA, United States), the plasmids were purified, and sequencing was performed to ensure their validity. The TaKaRa MutanBEST Kit (TaKaRa) was employed to produce plasmids that were specifically mutated for EBS.

### 2.5 Dual luciferase activity assay

The HEK293T and KGN cell types were cultured in 12-well plates for 24 h. Firefly and Renilla luciferase activities were quantified using a dual-luciferase assay kit and a microplate luminometer. Following the procedures described in our previous work, we carried out the transference of plasmids and measured luciferase activity (Abdurahman et al., 2019).

### 2.6 Bioinformatics analysis

The NCBI database BLAST tool was utilized to find homologous sequences. Use Promoter 2.0 Prediction Server online software to predict the promoter region. Forecast of transcription factor binding sites using online software (http://jaspardev.genereg.net).

### 2.7 Western blot

The total protein content of granulosa cells was extracted and measured using conventional techniques as well as the BCA Kit from Pierce, located in Shanghai, China. The procedure for Western blotting was carried out according to the previously outlined method (Abdurahman et al., 2022). For Western blotting the primary antibodies employed were specific to BMPR1B (Abcam, ab155058, Cambridge, United Kingdom, diluted to1:1500)and GAPDH (Abcam, ab9482, diluted to1:1500), and the secondary antibodies used were anti-goat and anti-mouse IgG (diluted to1:1500; Origene, Herford, Germany). ImageJ software was utilized to perform the examination of the bands.

### 2.8 Apoptosis analysis

The investigation into granulosa cell apoptosis utilized the Annexin V-FITC/propidium iodide apoptosis kit (KeyGene, Nanjing, China), following the manufacturer’s specified protocol. Flowjo software (version 7.6, Stanford University, Stanford, CA, United States) facilitated the calculation and analysis of the apoptosis rate in granulosa cells.

### 2.9 Statistical analysis

Statistical analyses were conducted to evaluate differences between groups using suitable parametric tests. The Student’s t-test was used for comparisons of two groups. For comparisons involving three or more groups, a one-way analysis of variance (one-way ANOVA) was performed. All analyses were carried out in SPSS version 27.0 (SPSS Inc., Chicago, IL, United States), and statistical significance was defined as *p ≤ 0.05.

## 3 Results

### 3.1 Sequence analysis of the regulatory region of the BMPR1B gene in hu sheep

To investigate the transcriptional regulation of the BMPR1B gene in Hu sheep, we performed PCR amplification targeting a 1.5 kb sequence upstream of the 5'-UTR within the 5′regulatory region. The base composition analysis found that the contents of each base in the sequence were 29.4% (A), 34.6% (T), 17.3% (C), and 18.7% (G), of which the AT content was 64% and the CG content was 36%, It can be seen that the C + G content is relatively low, See Supplementary Figure S1 for the detailed analysis (Supplementary Figure S1).

### 3.2 Identification of the essential promoter region of the ovine BMPR1B gene

**FIGURE 1:**
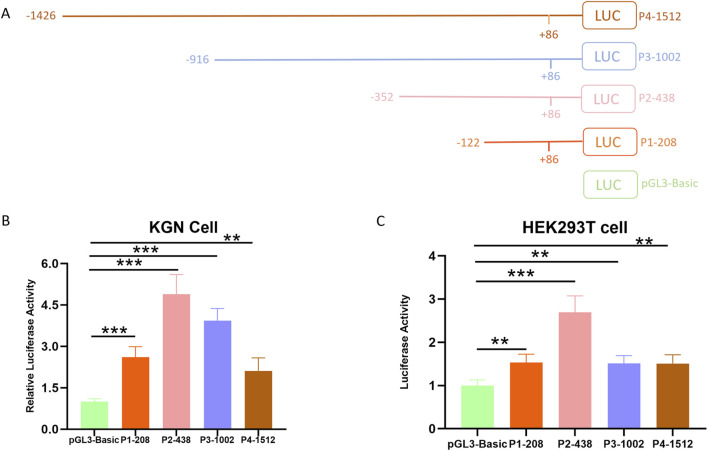
Determining the essential promoter area of the ovine BMPR1B gene. **(A)** The diagrammatic representation of the deletion constructs. **(B–C)** Luciferase assay. KGN cells **(B)** and HEK293T cells **(C)** were transfected with the deletion constructs, and the luciferase activity was evaluated through a Dual-Luciferase Reporter Assay System. The Bars show the average ±SEM derived from no fewer than three experiments. **p ≤ 0.01,***p ≤ 0.001.

The essential promoter, containing the smallest sequence necessary for precise initiation of transcription, is vital for controlling gene expression as it offers binding sites for RNA polymerase II and general transcription factors (Juven-Gershon et al., 2008). To identify the essential promoter region of the Hu sheep BMPR1B gene, we constructed four deletion vectors: -122/+86,-352/+86,-916/+86, and-1426/+86. Afterward, the luciferase reporter vector pGL3-basic was employed for the insertion process. This led to the successful creation of four deletion constructs, which were designated as P1-208, P2-438, P3-1002, and P4-1512 (Figure 1A). KGN cells, a type of granulosa cell line, along with HEK293T cells, were subjected to transfection with deletion constructs. Results from the dual-luciferase reporter assay indicated that the four deletion constructs were notably greater than that of the control group, with plasmid P2-438 treated KGN and HeLa cells exhibiting significantly elevated luciferase activity compared to the other three plasmid-treated KGN (Figure 1B) and HEK293T cells (Figure 1C). Overall, the data revealed that the promoter region located between −438 and −208 nucleotides, acts as the essential promoter area for the ovine BMPR1B gene.

### 3.3 Scrutiny of the essential promoter domain of the BMPR1B gene in sheep

**FIGURE 2:**
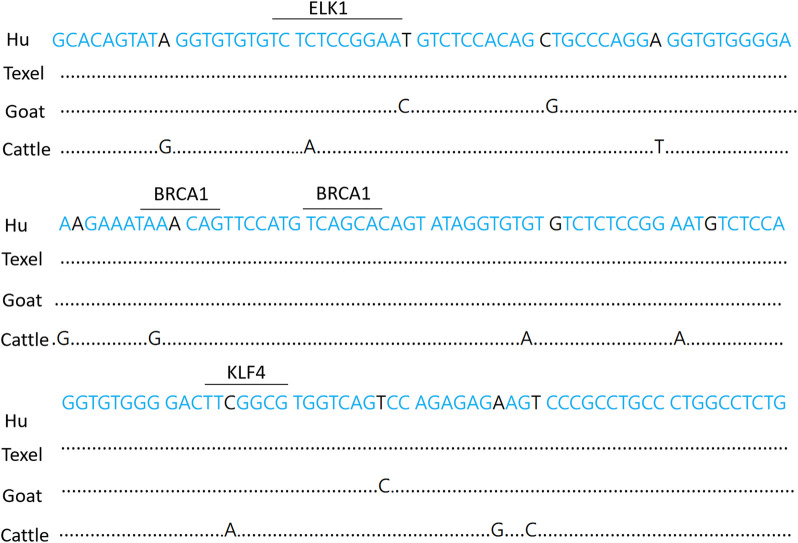
Hu sheep BMPR1B gene essential promoter sequence. The underlined sections represent potential transcription factor binding sites. Dots designate missing nucleotides.

The essential promoter of the BMPR1B gene in Hu sheep is composed of 72 A (31.3%), 60 T (26%), 48 C (21%), and 50 G (21.7%) nucleotides. There is a 99% match between the nucleotide sequence of the essential promoter and that of Texel sheep. The transcription factor binding sites within the promoter area of the BMPR1B gene in Hu sheep were predicted and examined using the online tools TRANSFAC (https://genexplain.com/transfac-product) and JASPAR (https://jaspar.elixir.no/). Findings indicated that numerous transcription factor binding locations exist in the region of the essential promoter. For instance, ELK1, BRCA1(Breast cancer 1), KLF4, Sp1, and more (Figure 2).

### 3.4 ELK1 regulates BMPR1B transcriptional activity in sheep ovary granulosa cells

**FIGURE 3:**
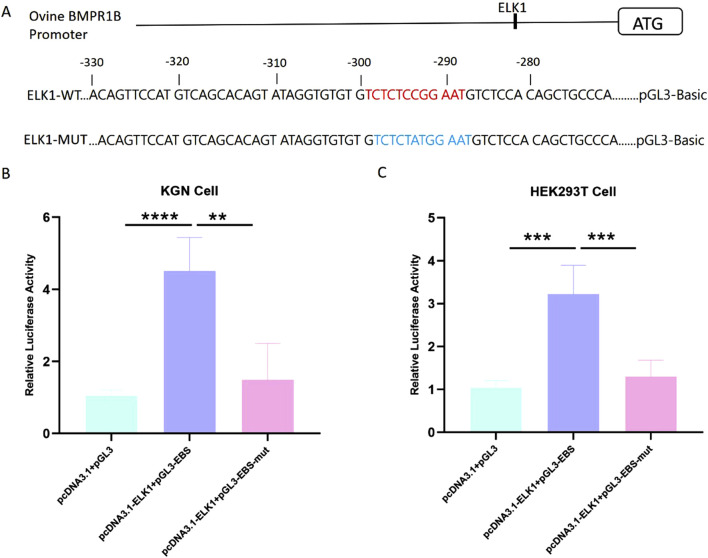
The gene BMPR1B in Hu sheep is regulated in its transcriptional activity by the presence of ELK1. **(A)**ELK1-binding site (EBS) and the essential promoter’s luciferase reporter constructs feature both wild-type and mutated ELK1-binding sites. **(B–C)**The effect of ELK1 overexpression on luciferase activity of the wild-type and mutant-typed promoter in KGN cells **(B)**and HEK293T cells **(C)**. **p≤ 0.01, ***p≤ 0.001, ****p< 0.0001.

**FIGURE 4:**
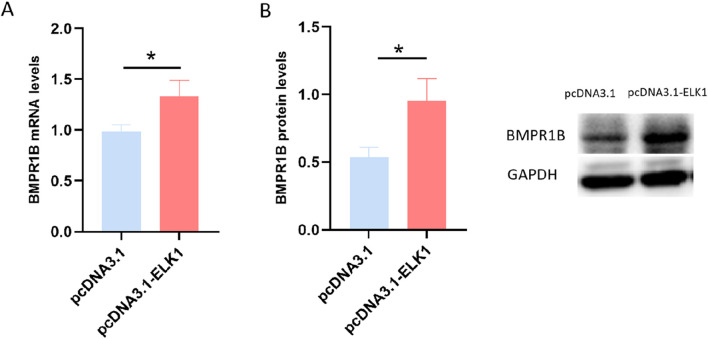
The influence of ELK1 on the expression of BMPR1B was assessed in ovine granulosa cells, where levels of BMPR1B expression were measured after transfection with the pcDNA3.1-ELK1 plasmid. **(A)** mRNA levels. **(B)** Protein levels, *p ≤ 0.05.

Studies have shown that transcription factor ELK-1 plays a core transcriptional regulatory role in reproductive function by regulating the cell proliferation and cell differentiation of many types of cells (Quintero-Barceinas et al., 2021; Jiang et al., 2020). To date, the influence of ELK1 on the transcriptional management of BMPR1B remains unreported. Significantly, the ELK1 motif was found within the essential promoter area of the Hu sheep BMPR1B gene. (Figure 4A). To explore the function of ELK1 as a regulator of transcription for the BMPR1B gene, wild-type, and mutant promoter region luciferase reporter vectors were constructed using a transcription factor ELK1 binding site. We constructed wild-type and mutant-type ELK1 binding site vectors using the BMPR1B promoter sequence. In addition, we constructed an ELK1 overexpression vector using the ELK1 coding sequence (Figure 3A). To investigate the potential regulatory role of the transcription factor ELK1 on the essential promoter activity of the BMPR1B, we created a luciferase reporter vector for the essential promoter and performed co-transfection with the ELK1 overexpression vector, pcDNA3.1-ELK1, in KGN and HEK293T cells. Results demonstrated that the elevated expression of ELK1 notably improved promoter activity BMPR1B gene in both KGN and HEK293T cell lines (Figures 3B,C). These findings indicate that ELK1 increases the promoter activity associated with the BMPR1B gene.

### 3.5 ELK1 enhances BMPR1B gene expression in ovarian granulosa cells

To investigate the influence of ELK1 on BMPR1B gene expression in sheep ovarian granulosa cells, we conducted a transfection of pcDNA3.1-ELK1 into cultured sheep ovary granulosa cells, followed by qRT-PCR analysis to measure BMPR1B mRNA levels. The findings revealed a substantial increase in BMPR1B gene mRNA levels in granulosa cells of the ELK1 overexpression group compared to the control group (Figure 4A). Moreover, Western blotting analysis revealed a considerable rise in BMPR1B protein concentrations in the granulosa cells of sheep ovaries in the ELK1 overexpression cohort as opposed to the control cohort. (Figure 4B). The data points to ELK1 playing a role as a transcriptional activator for the BMPR1B gene in the granulosa cells of ovine ovaries.

### 3.6 Transcription factor ELK1 inhibits apoptosis of sheep ovary granulosa cells

**FIGURE 5:**
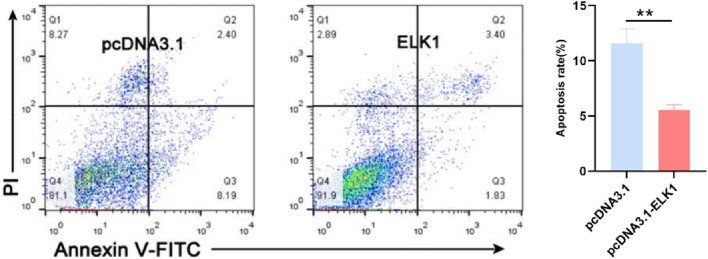
ELK1 inhibits the ovine granulosa cell apoptosis ELK1 suppresses granulosa cell apoptosis. ELK1 expression vector pcDNA3.1- ELK1 was transfected into the ovine granulosa cells, and cell apoptosis rate was detected using FACS. **p ≤ 0.01.

Studies have shown that BMPR1B functions to inhibit apoptosis in granulosa cells of sheep (Abdurahman et al., 2019). To explore the role of ELK1 in the process of apoptosis within sheep ovarian granulosa cells, we transfected pcDNA3.1-ELK1 into granulosa cells derived from sheep ovaries that were maintained *in vitro*. Subsequently, We carried out an assessment of the apoptosis rate in these cells through a fluorescence-activated cell sorting (FACS) method. The data presented in Figure 5 reveal a marked reduction in apoptotic cell death (*P* ≤ 0.01) in ELK1-overexpressing ovarian granulosa cells compared to the control group. This indicates that the transcription factor ELK1 can suppress apoptosis in sheep ovary granulosa cells, aligning with the known function of BMPR1B in managing the process of apoptosis in ovarian granulosa cells.

## 4 Discussion

In sheep genetics, the regulation of reproductive traits is heavily influenced by the important BMPR1B gene (Akhatayeva et al., 2023). A study revealed that high-fertility sheep had significantly higher transcription levels of the BMPR1B gene in ovarian tissue compared to low-fertility sheep (Xu et al., 2010; Zhong et al., 2024). However, there is a lack of research on the transcription of this gene in sheep ovarian tissue. We identified the essential promoter within the 5′regulatory region of BMPR1B in Hu sheep and analyzed its sequence. Luciferase reporter assays identified the region from −438 bp to −208 bp relative to the essential promoter of the BMPR1B gene as a critical regulatory element. Our results showed that segment P1 also exhibits luciferase activity. However, P2 exhibited robust and stable activity across both cell lines, with a significantly higher magnitude than P1. Additionally, we identified some important transcription factors within the sequence of P2, which may further enhance its activity. It has been established for a while that certain genes possess multiple promoters (Ayoubi and Van De Ven, 1996). Recent research also indicates that a significant portion of the human genome is transcribed from both DNA strands. Additionally, many human genes possess multiple promoters, enabling transcription under various cellular conditions (Kimura et al., 2006). As for P1, We will investigate its activity in more detail in our further study. Such findings may establish a groundwork for subsequent studies focused on the regulation of its transcription. Interestingly, we discovered numerous binding sites for transcription factors (TFBSs) located in the central promoter region. Certain transcription factors, including ELK1 (Fan et al., 2009; Pan et al., 2021; Zhang and Zhang, 2020), KLF4 (Miao et al., 2023), BRCA1 (Turan and Oktay, 2020), and Sp1 (Cai et al., 2020; Zhou et al., 2023), play a significant role in female and animal reproductive processes, akin to the function of BMPR1B. The transcription factor Sp1, a member of the Sp/KLF family, plays a crucial role in ovarian function by modulating the expression of various genes associated with female reproduction, including CYP11A1 (Ahlgren et al., 1999; Lo et al., 2017), IRS-2 (Kaur et al., 2016), and RGS (Ivanenko et al., 2022). It achieves this by directly interacting with a specific GC-rich motif located in the promoters of these genes within the mammalian ovary (Sogut et al., 2021; Martin-Malpartida et al., 2017; Sharrocks et al., 1997).

ELK1 is a key factor in regulating the transcription and biological functions of target genes (Besnard et al., 2011), impacting cell proliferation, differentiation, apoptosis, migration, and pathology (H et al., 2024). Our results revealed that ELK1 increases the transcriptional function of the BMPR1B gene by attaching to its promoter, which leads to the inhibition of apoptosis in the ovarian granulosa cells of sheep. In porcine ovary granulosa cells, β-defensin 3 (pDEFB103A) has been shown to enhance cell proliferation and migration through the ERK/ELK1 system (Liu et al., 2019). Additionally, in the human ovarian granulosa cell line KGN, hepatocyte growth factor (HGF) has been found to stimulate granulosa cell proliferation, suppress progesterone production, reduce phosphorylation of ERK1/2 and ELK1, and inhibit the expression of StAR, a crucial regulator of the progesterone synthesis pathway (Taniguchi et al., 2004). Furthermore, GnRH, which is synthesized and released by the pituitary gland, plays a role in regulating steroid hormone production, follicle maturation, and ovulation in the ovary through specific receptors (Bliss et al., 2010). Studies with *in vitro* cultured human luteal granulosa cells have demonstrated that GnRH exerts its effects via the MAPK pathway and activation of ELK1 (Kang et al., 2001). Similarly, Studies have demonstrated that melatonin can affect progesterone production in human luteal granulosa cells through an identical process (Fang et al., 2019). Our investigation indicated that the EBS site is located within the promoter area of the ovine BMPR1B gene, and we determined that ELK1 acts as a transcription factor that regulates the expression of BMPR1B in ovine granulosa cells. The primary mechanism through which ELK1 exerts its biological effects involves binding to the promoter regions of significant functional genes and influencing their transcription (Thiel et al., 2021; Odrowaz and Sharrocks, 2012). In stem cells and tumor cells, ELK1 promotes proliferation, and inhibits apoptosis and differentiation; in the nervous system, it participates in processes such as long-term memory (Daws et al., 2013). ELK1 levels are markedly high in the context of ovarian cancer cells, promoting cell proliferation and migration, and affecting chemotherapy resistance by regulating genes. For example, In human ovarian cancer that is resistant to paclitaxel, the expression of miR-134 is suppressed by ELK1, which increases TAB1 levels (Shuang et al., 2017). Moreover, the ongoing activation of the ERK/ELK1 pathway in ovarian cancer is discussed, particularly regarding its role in cell proliferation, and it is highlighted that inhibiting ERK1/2 can effectively curtail tumor expansion (Steinmetz et al., 2004). In mammalian granulosa cells, researchers have identified only a limited number of essential genes that regulate granulosa cell activities, which act as direct transcriptional targets of the transcription factor ELK1. Luteinizing hormone (LH) activates ELK1 phosphorylation through the EGFR-ERK1/2 signaling pathway, upregulating the RNA binding protein TTP, leading to the degradation of the meiotic arrest factor Nppc mRNA, thereby relieving the meiotic arrest of oocytes (Xi et al., 2021). This mechanism is crucial for preovulatory follicle development and haploid oogenesis in mammals. As a core member of the ETS family, the transcription factor ELK1 plays a key role in cell proliferation, differentiation, and stress response by integrating signaling pathways such as MAPK/ERK. Our study found that ELK1 overexpression can significantly upregulate the mRNA and protein levels of the sheep reproductive master gene BMPR1B, thereby further realizing the key role of the BMPR1B gene in animal reproduction. Functionally, ELK1 overexpression can significantly reduce the apoptosis of Hu sheep ovarian granulosa cells. Our laboratory’s previous studies demonstrate that miR-1306 causes apoptosis in sheep granulosa cells through its action on the BMPR1B gene (Abdurahman et al., 2022).

## 5 Conclusion

In conclusion, our findings indicate that the essential promoter region of the Hu sheep BMPR1B gene includes multiple regulatory elements. Furthermore, ELK1 functions as a transcription regulator, Increasing the levels of BMPR1B and lowering the apoptosis rate in ovarian granulosa cells, which in turn affects BMPR1B expression levels in granulosa cells. These findings enhance our comprehension of the mechanisms behind granulosa cell apoptosis, the development and progression of ovarian follicles, and female reproductive biology in mammals.

## Data Availability

The raw data supporting the conclusions of this article will be made available by the authors, without undue reservation.
