# Supplementary figures and images for: ELK1 regulates BMPR1B transcriptional activity in ovine granulosa cells
Source: Front Cell Dev Biol. 2025 Jul 1;13:1623135. doi: 10.3389/fcell.2025.1623135 (PMC12259578; doi:10.3389/fcell.2025.1623135)

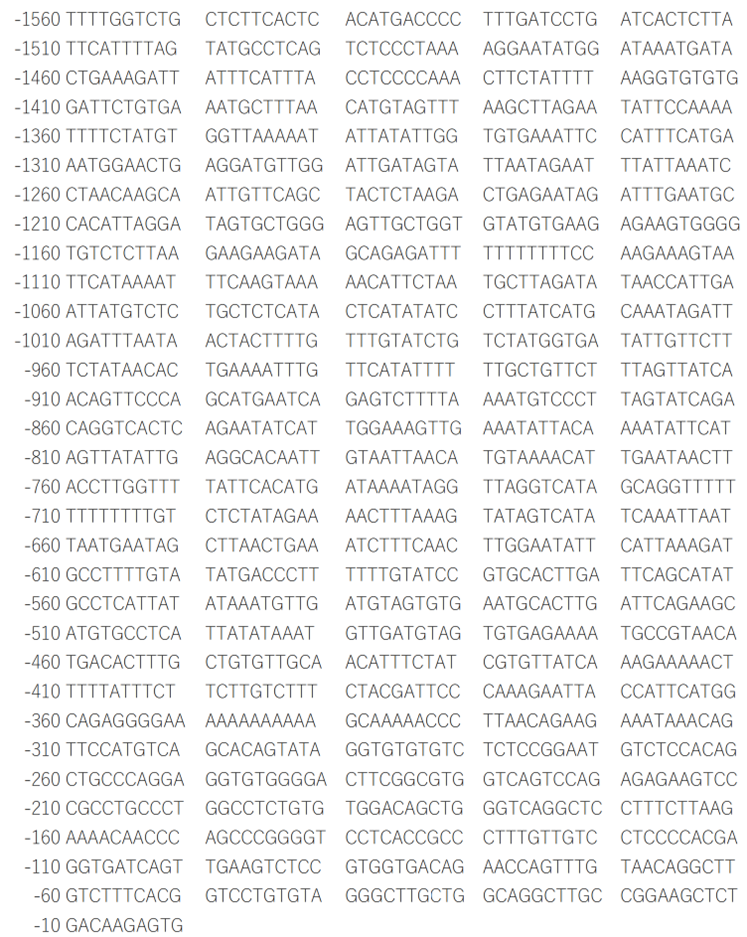


Figure S1.The 5'regulating region sequence of BMPR1B in Hu sheep

Supplement: Supplementary file 1 [file Table1.docx]
